# Functional Change in Experimental Allodynia After Glutamate-Induced Pain in the Human Masseter Muscle

**DOI:** 10.3389/froh.2020.609082

**Published:** 2020-11-23

**Authors:** Akiko Shimada, Abdelrahman M. Alhilou, Peter Svensson, Malin Ernberg, Nikolaos Christidis

**Affiliations:** ^1^Department of Geriatric Dentistry, Osaka Dental University, Osaka, Japan; ^2^Division of Oral Diagnostics and Rehabilitation, Department of Dental Medicine, Karolinska Institutet, Huddinge, Sweden; ^3^Scandinavian Center for Orofacial Neurosciences (SCON), Huddinge, Sweden; ^4^Department of Restorative Dentistry, College of Dentistry, Umm Al-Qura University, Mecca, Saudi Arabia; ^5^Section of Orofacial Pain and Jaw Function, Department of Dentistry and Oral Health, Aarhus University, Aarhus, Denmark; ^6^Faculty of Odontology, Malmö University, Malmö, Sweden

**Keywords:** masseter myalgia, allodynia, glutamate, nerve growth factor, temporomandibular disorders

## Abstract

**Background:** Glutamate, as well as nerve growth factor (NGF), is involved in nociception from peripheral tissues, such as muscles. However, the potential interaction between glutamate and NGF still remains unclear. This study investigated the interaction between glutamate-induced masseter muscle pain and NGF-induced allodynia on pain perception and jaw function in healthy individuals, and any possible sex differences in the response.

**Materials and Methods:** Thirty pain-free adult participants (15 men and 15 women, mean age ± *SD*: 24 ± 4 years) participated in this study consisting of three sessions (Day 0, Day 3, and Day 4). NGF (5 μg/mL, 1.0 mL) was injected into the masseter muscle on Day 0 to induce muscle allodynia. On Day 3, glutamate (1M, 0.2 mL) was injected into the same masseter muscle. Before and after injections on Day 0 and 3, and post-injection (Day 4), spontaneous pain, temporal summation pain, as well as functional pain and fatigue in response to chewing were assessed with validated scales, and the pressure pain threshold (PPT) was recorded.

**Results:** Spontaneous pain intensity was significantly higher after glutamate than NGF (*P* < 0.001). PPTs, temporal summation pain and functional measures were all reduced 3 days after NGF injection (*P*'s < 0.001). Injection of glutamate on Day 3 did not further affect PPTs or temporal summation pain and there were no sex differences in the effects (*P* > 0.189). Chewing pain (*P* = 0.022) and fatigue increased after glutamate injection to a higher degree in the women than men (*P* = 0.037).

**Conclusion:** Taken together, while glutamate injected into the NGF-sensitized muscle was painful, it did not alter muscle tenderness in women vs. men. However, pain and fatigue evoked by jaw function were higher in women after glutamate injection. This suggest that sex differences reported for masseter myalgia, mimicked by glutamate and NGF mediated pain in this study, may be greater for measures of perceived jaw function, which should be considered in a clinical evaluation.

## Introduction

TMD is a collective term embracing chronic musculoskeletal pain conditions affecting the temporomandibular joint (TMJ) or the jaw muscles and their associated structures [1]. The prevalence of chronic TMD pain in the population is 10–15% and it is about 1.5 to 2 times higher in women than men [1]. TMD myalgia is the most common diagnosis. The pathophysiology behind TMD myalgia is to great extent unknown [2], but mechanical overloading and ensuing relative hypoxia or ischemia of the muscles are suggested to be involved. This may lead to an increased muscle tonus and a cascade of biochemical events including release of algesic substances, such as glutamate, serotonin, and neuropeptides, but possibly also to presently unknown algesic peptides and proteins [3]. These algesic substances activate peripheral sensory afferents, which start a cascade of intracellular and extracellular events sensitizing the neuron and causing pain. Some inflammatory mediators are further reported to induce neuroplastic changes in the brainstem, central sensitization, which is proposed to contribute to muscle tenderness (i.e., allodynia and hyperalgesia).

To understand the pathophysiology of TMD myalgia, experimental pain models may be used, for example injection of algesic substances [4]. Injection of glutamate into the masseter muscle of healthy participants evokes experimental muscle pain with features comparable to the pain and mechanical sensitization reported by patients with TMD myalgia [5, 6]. Many studies conclude that this is due to activation of peripheral N-methyl-d-aspartate (NMDA) receptors located in the muscle tissue [6, 7]. Therefore, experimental studies based on glutamate injection into the masticatory muscles provide an appropriate model for studying different aspects of TMD myalgia [6]. However, the effect on pain and mechanical sensitization is short-lasting why the model more represents an acute experimental pain model.

Neurotrophins, such as nerve growth factor (NGF), are potent modulators of nociceptive information and thus, may play a role in chronic pain. NGF binds to the high-affinity tyrosine kinase receptor A (TrkA) that are expressed on sensory neurons and thus, sensitize the neuron [8]. NGF injected into the masseter muscle of pain-free volunteers did not provoke pain, but mechanical allodynia that lasted up to seven days [9]. Temporal summation, induced by repetitive mechanical stimuli has been shown to be affected by NGF injection into the muscle [10]. These results indicate that NGF injection may be a useful model for mechanical muscle allodynia [11].

A few studies have explored the interaction between NGF and other algesic substances. One study investigated the effects of glutamate injection into the masseter muscle in combination with NGF-induced mechanical sensitization in healthy men [12]. Even though no significant interaction between glutamate and NGF was observed in terms of pain intensity, injection of glutamate into the masseter muscle pretreated with NGF increased the area of pain spread. Another study reported that injection of hypertonic saline 1 day after NGF injection into the tibialis anterior muscle induced more pain than injection of hypertonic saline 1 day after isotonic saline injection, but only in men [13]. Men also scored the pain intensity higher than women after hypertonic saline injection into the muscle pretreated with NGF. This was unexpected since many previous studies have reported sex differences in pain, but in the opposite direction [14, 15]. For example, perceived pain intensity from temporal summation is reported to be higher in women [14, 16]. Moreover, the sensory perception during functional movements in a sensitized muscle might differ between sexes. However, this still remains unclear.

The aim of this study was to investigate the additive effect of glutamate injection into the NGF-sensitized masseter muscle on pain perception and jaw function in healthy participants, and differences in the response to the experimental pain between men and women. We hypothesized that the interaction between NGF and glutamate would increase mechanical muscle sensitivity, as well as pain and fatigue during functional movements, and that the effect would be greater in women than in men.

## Materials and Methods

### Participants

Fifteen healthy men and 15 age-matched healthy women (mean age ± *SD*: 24 ± 4 years old) were recruited to this study by ads posted around the campus at Aarhus University and through an online volunteer recruiting system (https://aucobe.sona-systems.com/). Exclusion criteria were a diagnosis of myalgia or myofascial pain with pain referral according to the Diagnostic Criteria for TMD (DC/TMD) [17], current facial pain or palpatory tenderness of the masseter muscle, continuous use of medication except birth control pills, and use of analgesic or anti-inflammatory medication during the 24 h preceding the experiment. Prior to the experiment, informed consent was obtained from all the participants. The study was approved by the local ethics committee (approval No. 1-10-72-199-15, Aarhus County, Denmark) and conducted in accordance with the Helsinki Declaration II. This study was not designed as a clinical trial, because there was no control group or sample randomization. However, this study could be characterized as an observational study where pain-related parameters were evaluated in an intervention model induced by the injections of glutamate or NGF.

### Study Design

**Figure 1:**
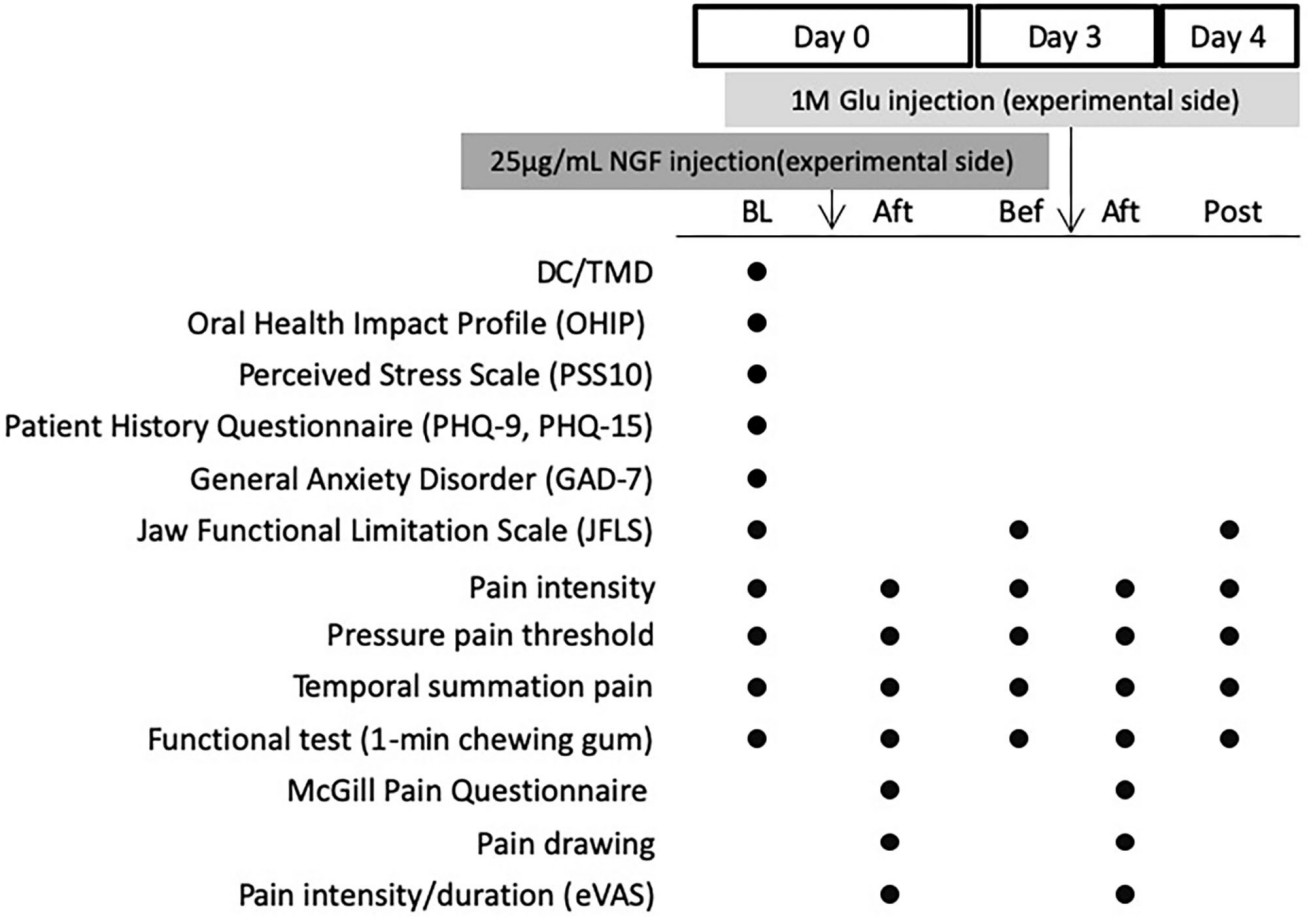
Experimental Design showing assessments done before and after injection of nerve growth factor (NGF, 25 mg/mL) on Day 0 and glutamate (Glu, 1M) on Day 3 into the masseter muscle of 30 healthy volunteers. BL, Baseline (before any injection); Bef, before injection; Aft, After injection. Post: On Day 4. DC/TMD, clinical examination according to the Diagnostic Criteria for Temporomandibular Disorder; eVAS, electronic visual analog scale.

This study is part of a larger project in which the interaction between NGF and glutamate on the expression of pain-related molecules is investigated in muscle biopsies (results presented elsewhere). The design of this study is shown in Figure 1. The experiment included three sessions (Day 0, 3, and 4). On Day 0 NGF was injected and on Day 3 glutamate was injected. During both days, outcome measures were assessed both before and after injections. Day 4 included assessments only.

After the absence of any current facial pain and TMD pain diagnosis (DC/TMD) [17] had been confirmed at Day 0, the participants were asked to complete the Symptom Questionnaire of the DC/TMD Axis II questionnaire for assessment of TMD symptoms [17], the Perceived Stress Scale (PSS-10) [18], the Patient History Questionnaire (PHQ-9 and PHQ-15), and the General Anxiety Disorder Questionnaire (GAD-7) for emotional function [19, 20], and the Oral Health Impact Profile (OHIP-14) for life quality [21]. The Jaw Functional Limitation Scale (JFLS) was further completed [22].

Before injections on Day 0 (NGF) and Day 3 (glutamate), as well as on Day 4 pressure pain thresholds (PPT) and temporal summation pain were recorded, and a functional test was performed [23]. As soon as the needle for the injection was inserted on Day 0 and Day 3, participants were asked to continuously assess the pain intensity until the pain had declined or maximum 10 min [24, 25]. Five minutes after the injections recording of PPT and temporal summation pain were repeated as well as the functional test. In addition the McGill Pain Questionnaire (MPQ) was completed [26] and the painful area evoked by the injection mapped on pain drawings [6]. On Day 0 (contralateral side) and Day 4 (ipsilateral side as NGF and glutamate injections) a microbiopsy [27] was taken from the masseter for later analyses of algesic molecules (results to be presented elsewhere).

### Injections

Injections were given into the right masseter muscle on both occasions. The bulkiest point of the masseter belly was used for injections (i.e., ~2 cm above mandibular border and 2 cm anterior of the ramus boarder [9]). At Day 0, sterile NGF (0.4 mL, 25 μg/mL, Skanderborg Pharmacy, Denmark) was injected [9], and on Day 3, when the effect of NGF is most prominent [23], sterile glutamate solution (0.2 mL 1M, Skanderborg Pharmacy, Denmark) [25].

### Pain Evoked by Injections

Pain intensity was assessed with an electronic visual analog scale (eVAS) ranging from 0 (no pain) to 10 (worst pain imaginable) [28]. The mean pain intensity during 10 min was calculated and used in the analyses. For assessment of pain spread the participants were asked to draw the painful area 5 min after each injection on a picture that represented a lateral view of the face (both sides). The pain drawing area was digitized (Image J 1.50i) and expressed in arbitrary units [29].

The MPQ is composed of 80 adjectives that relate to pain. The participants chose those adjectives that best describe their pain. The adjectives are divided into sensory, affective, evaluative, and miscellaneous sections and each section is graded according to severity. The sum of the scores for the four categories can be calculated as the Pain Rating Index (PRI), which was used in this study [26].

### Pressure Pain Thresholds and Temporal Summation

PPT was recorded bilaterally at two points [injection point (P1) and a point 1 cm superior to the injection point (P2)] of the masseter muscle with an electronic algometer (Somedic Sales AB, Hörby, Sweden) [30, 31]. Each recording of PPT was performed three times at each site and the mean of the three recordings used for the later analysis.

Temporal summation was evoked with a 1.0 kg palpometer (Palpeter®, Sunstar Suisse SA, Switzerland) [32]. The stimulus was applied 10 times at the masseter muscle [injection point (P1)] for 1 with 2 s interval and pain intensity was recorded after the 1st, 5th, and 10th stimulation on a 0–10 numeric rating scale (NRS) with the same endpoints as the eVAS.

### Jaw Function

The JFLS was completed by each participant in the beginning of each session (Day 0, Day 3, and Day 4). It consists 20 questions that are scored 0–10. The total sum was calculated [22].

To asses pain and fatigue evoked by jaw function, a functional test was performed. The participants were asked to chew a gum (V6, 2 piece, Fertin Pharma, Vejle, Denmark) during 1 min and thereafter to score the pain and fatigue evoked [33]. The pain intensity was assessed with 0–10 NRS and the fatigue level with Borg's Rating of Perceived Exertion scale (RPE) [34]. This scale ranges from 6 (no exertion at all) to 20 (maximal exertion).

### Statistics

A sample-size calculation performed before the study showed that with 15 participants per group we would be able to detect a 10 (*SD* 9) % difference in means with a power of more than 80% at a significance level of 5%.

The normality of the data was tested with D'Agostino & Pearson test. Descriptive data are presented as mean ± standard deviation (*SD*) or median (interquartile range, IQR) depending on type of data and normality.

Among scores of the questionnaires taken only at baseline (PSS-10, PHQ-9, PHQ-15, GAD-7, and OHIP), Mann-Whitney test for non-parametric data was used to determine if there was a significant influence of sex.

For analysis of pain-related parameters after injections such as VAS, MPQ (PRI scores), and pain drawing areas, two-way ANOVAs were used. The main factors for the ANOVA were sex (2 levels: men and women) and substance (2 levels: NGF and glutamate). Furthermore, scores of JFLS were analyzed with 2-way ANOVA with repeated measurements whose main factors were sex (2 levels: men and women) and day [3 levels: Day 0 (baseline), Day 3, Day 4].

Two-way ANOVAs with repeated measurements were also used for analysis of PPT and temporal summation pain on injected side and control side, as well as pain and fatigue level after chewing. For PPT and temporal summation pain the average of assessments from the two points (P1 and P2) were used. Main factors of the ANOVAs were sex (2 levels: men and women), and time [5 levels: Day 0 (baseline and after NGF injection), Day 3 (before and after glutamate injection), and Day 4 (post-session)]. When appropriate, *post-hoc* test (Holm-Sidak's multiple comparisons) was applied.

Prism 8 (version 8.4.3; GraphPad Software, Inc.) was used for statistical analyses. *P* < 0.05 was considered significant.

## Results

The baseline characteristics of the participants are shown in Table 1. None of the participants had any facial pain or tenderness to palpation of the masseter muscle at baseline and none of them were diagnosed with pain-related TMD (i.e., myalgia, arthralgia, or headache attributed to TMD. The women reported higher levels of depression (PHQ-9), anxiety (GAD-7), and non-specific physical symptoms (PHQ-15) than the men, with slightly increased values compared to normal. Stress level (PSS-10) and life quality did not differ between sexes. All participants participated in all session (i.e., there were no drop-outs).

**Table 1:** Baseline assessment of emotional function and quality of life.

		**All**		**Men**		**Women**		**Sex difference**
		***N*** **=** **30**		***N*** **=** **15**		***N*** **=** **15**		***P*-values**
OHIP	(0–56)	1	(0, 5)	2	(0, 11)	1	(1, 3)	0.94
PSS10	(0–40)	12	(8, 16)	10	(7, 17)	13	(11, 16)	0.23
PHQ-9	(0–27)	4	(1, 7)	1	(0, 6)	4	(3, 7)	0.03^*^
PHQ-15	(0–30)	4	(1, 6)	2	(0, 4)	5	(4, 7)	<0.01^*^
GAD-7	(0–21)	2	(0, 3)	0	(0, 2)	2	(1, 7)	0.03^*^

*The scores of each questionnaire is shown as median and interquartile range, IQR*.

* *Indicates a significant difference between sexes*.

*P < 0.05*.

*OHIP, the Oral Health Impact Profile; PSS, the Perceived Stress Scale; PHQ, the Patient History Questionnaire; GAD, the General Anxiety Disorder Questionnaire*.

### Effect of Experimental Pain

**Figure 2:**
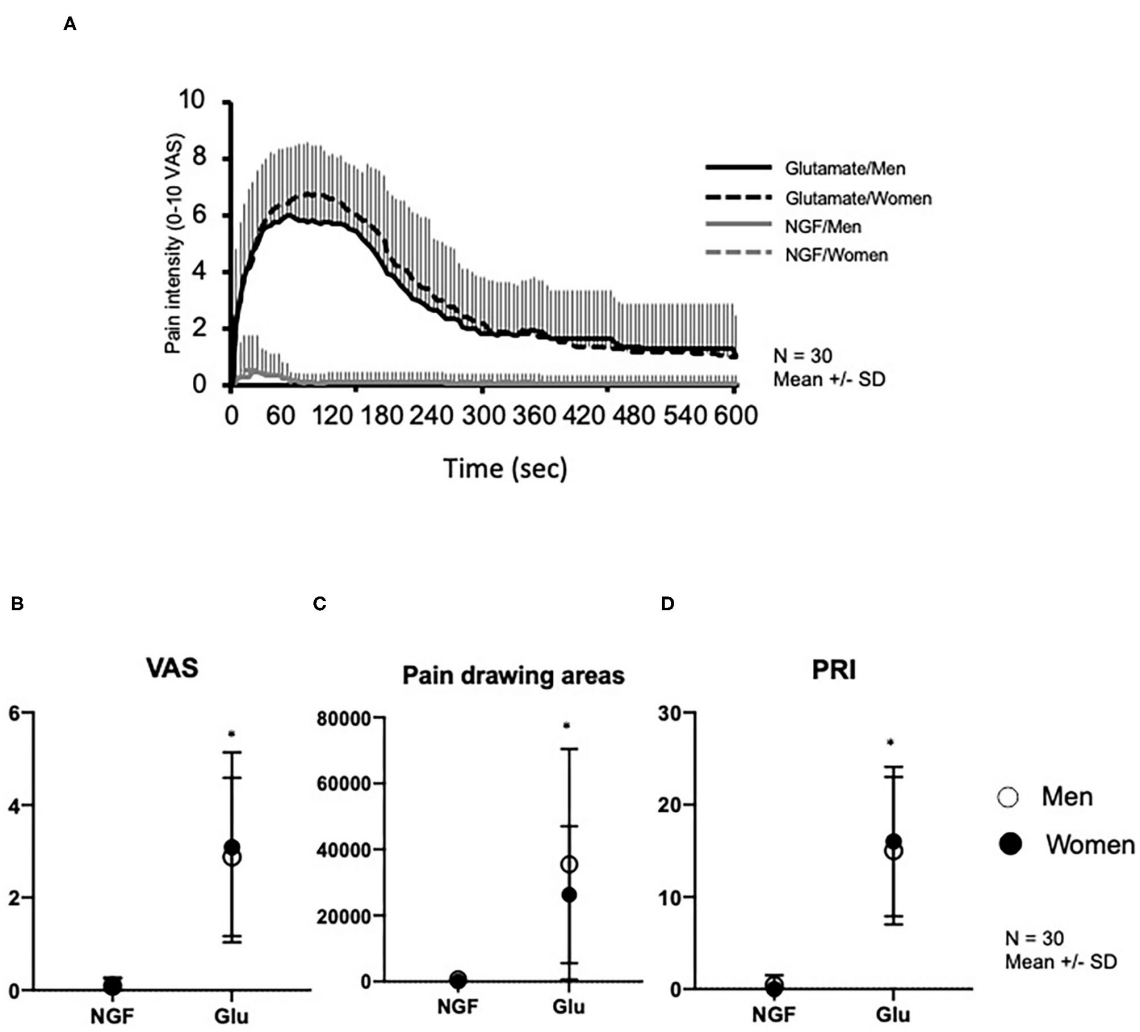
Pain variables assessed after injection of nerve growth factor (NGF) on Day 0 and glutamate (Glu) on Day 3 into the masseter muscle of 30 healthy volunteers. **(A)** Change of pain intensity assessed with 0–10 electronic visual analog scale (eVAS). **(B)** Maximum pain intensity retrieved from the eVAS. **(C)** Pain drawings area (mm^2^). **(D)** Pain Rating Index (PRI) assessed with McGill Pain Questionnaire. *indicates a significant difference between substances (*P* < 0.05). Open circles show men, whereas solid circles show women. The data is shown as mean and standard deviation (*SD*).

Figure 2A shows changes in pain intensity (eVAS) after NGF and glutamate injections. There was a significant main effect of substance on pain intensity (*F* = 70.25, *P* < 0.001), PRI score of MPQ (*F* = 107.4, *P* < 0.001), and pain drawing areas (*F* = 33.93, *P* < 0.001), but no significant effect of sex (*F* < 0.885, *P* > 0.351) or interaction between substance and sex. Both sex groups reported significantly higher maximum pain intensity (Figure 2B), PRI scores (Figure 2C), and larger pain drawing area (Figure 2D) after glutamate than NGF injection.

### Pressure Pain Thresholds and Temporal Summation Pain

**Figure 3:**
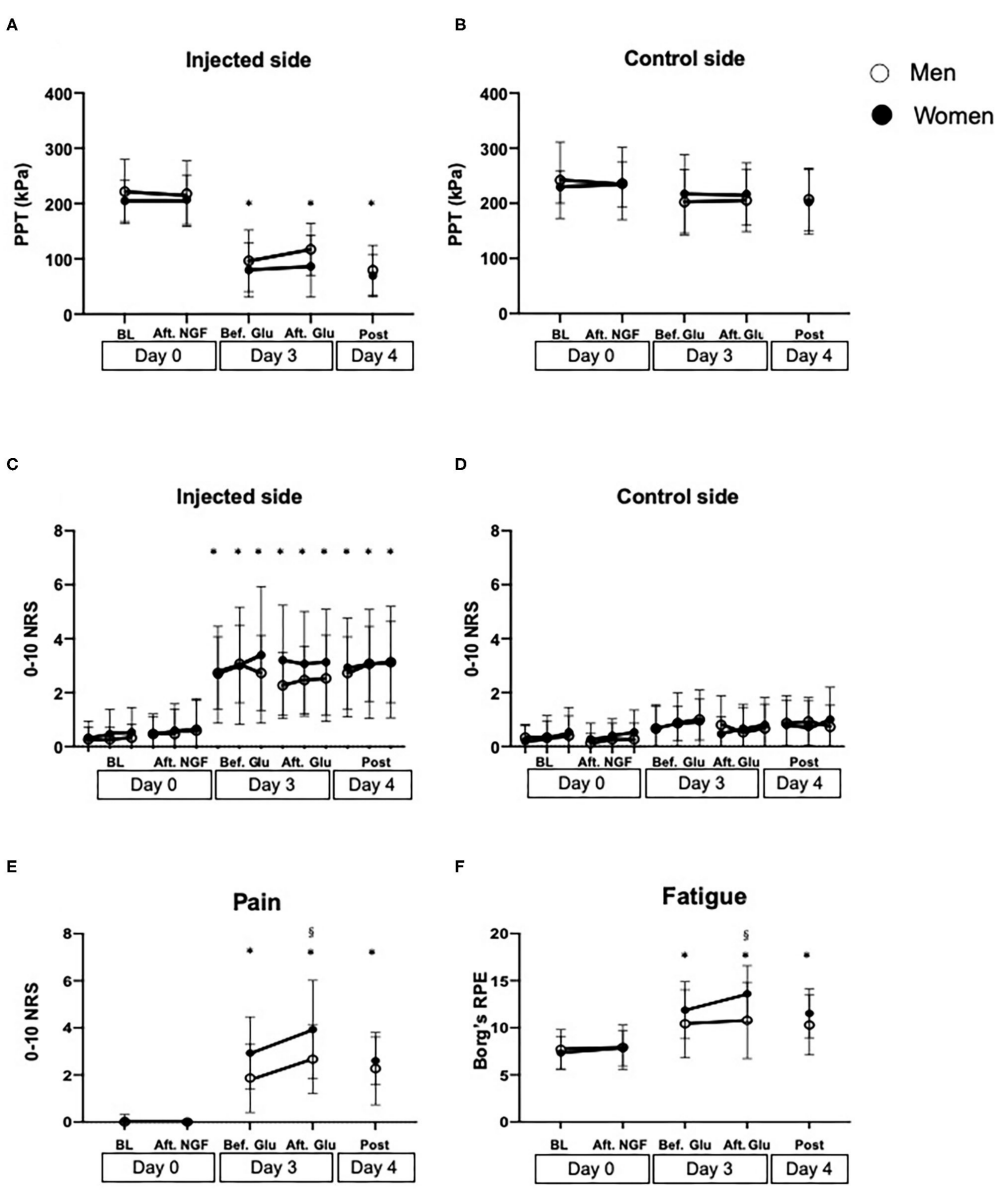
Measures of muscle sensitization assessed before (BL) and after injection of nerve growth factor (Aft. NGF) on Day 0, before and after glutamate (Bef. Glu and Aft. Glu, respectively) on Day 3, and on Day 4 (Post) into the masseter muscle of 30 healthy volunteers. **(A)** Pressure pain thresholds (PPT, kPa) on the injected side and **(B)** on the control side. **(C)** Temporal summation pain (0–10 numeric rating scale, NRS) on the injected side and **(D)** on the control side. **(E)** Pain intensity (NRS) and **(F)** fatigue (Borg's RPE scale) after a 1-min chewing test. *indicates a significant difference compared to baseline in both sexes (*P* < 0.05). ^§^indicates a significant difference between sexes. Open circles show men, whereas solid circles show women. The data is shown as mean and standard deviation (*SD*).

Although there was no significant difference in PPTs between the injected and control sides at baseline (Day 0) (Men: *P* = 0.996, Women: *P* = 0.998), PPT values in the injected side significantly decreased on Day 3 (before glutamate injection), compared to the control side in both men and women (Men: *P* < 0.001, Women: *P* < 0.001). For PPTs on the experimental side, there was a significant effect of sex (*F* = 1.165, *P* = 0.039) with lower values in women. There was also an effect of time (*F* = 61.50, *P* < 0.001), but no interaction between sex and time (*F* = 0.310, *P* = 0.872). Holm-Sidak's *post-hoc* test showed that the PPTs were significantly lower at all the time points on Day 3 (before and after glutamate injection) and Day 4 compared to baseline (Day 0), but there were no significant differences between the other time points. On the control side, there was no significant effect of either sex (*F* = 0.013, *P* = 0.909) or time (*F* = 1.018, *P* = 0.401) and no interaction between them (*F* = 0.310, *P* = 0.871) (Figures 3A,B).

Significant effect of time was also shown for temporal summation pain on the experimental side (*F* = 42.21, *P* < 0.001), whereas there was no effect of sex (*F* = 2.964, *P* = 0.086) or any interaction (*F* = 0.346, *P* = 0.987). The *post-hoc* test showed that the pain intensity was significantly higher on Day 3 (before and after glutamate injection) and Day 4 (*P* < 0.020) compared to baseline (Day 0) in both groups, but there were no other significant differences between the other time points, in similarity with PPTs. There was no significant difference between sex at any time points (Figures 3C,D).

### Functional Test

Change in scores of the JFLS is shown in Table 2. The 2-way ANOVA showed a significant effect of time (*F* = 21.69, *P* < 0.001) but not sex (*F* = 0.044, *P* = 0.834), and no interaction between them (*F* = 0.344, *P* = 0.710). *Post-hoc* tests showed that the scores on Day 3 and 4 were significantly higher than on Day 0 (*P* < 0.001).

**Table 2:** Scores in the Jaw Functional Rating scale are shown in mean ± standard deviation (*SD*).

	**All**	**Men**	**Women**
	***N* = 30**	***N* = 15**	***N* = 15**
Day 0	1.4 ± 5.5	0.5 ± 1.6	2.3 ± 7.7
Day 3	25.2 ± 20.7	23.6 ± 22.9	26.9 ± 18.8
Day 4	19.6 ± 14.2	20.9 ± 15.3	18.2 ± 13.5

Sex (*F* = 7.813, *P* = 0.006) and time (*F* = 48.28, *P* < 0.001) showed significant main effects on chewing pain, whereas there was no significant interaction between them (*F* = 1.783, *P* = 0.136). *Post-hoc* test showed that both men and women showed increased chewing pain on Day 3, both before and after glutamate injection and post session compared to baseline Day 0 (*P* = 0.022) but there were no significant differences between any other time points (*P* < 0.078). Chewing pain was significantly higher in women than men after glutamate injection on Day 3 (*P* = 0.022) (Figure 3E).

Chewing fatigue showed a similar pattern as chewing pain with a significant effect of sex (*F* = 4.634, *P* = 0.033) and time (*F* = 16.39, *P* < 0.001), but there was no significant interaction between them (*F* = 1.564, *P* = 0.187). *Post-hoc* test showed that there was no significant change in fatigue in the men throughout the experimental sessions (*P* > 0.116), whereas chewing fatigue in women was significantly higher before and after glutamate injection on Day 3 than at baseline Day 0 (*P* = 0.001). Furthermore, chewing fatigue in women was significantly higher than in men after glutamate injection on Day 3 (*P* = 0.037) (Figure 3F).

## Discussion

This study demonstrated that experimental spontaneous acute muscle pain induced by local injection of monosodium glutamate into the NGF sensitized masseter muscle of healthy pain-free individuals of both sexes did not further alter PPTs or temporal summation pain. However, self-reported pain and fatigue level in response to a chewing task worsened after glutamate injection and this effect was more pronounced in women compared to men.

NGF injection into the masseter muscle did not evoke pain, but induced mechanical allodynia and hyperalgesia that peaked 3 days after the injection in this study. The effect of the NGF injection was shown as reduced PPTs, increased pain level during temporal summation, and increased pain and fatigue during jaw function. Similar effects are well-reported in the literature where an intermuscular NGF injection with the same concentration into the masseter muscle was administrated in men and induced long-lasting mechanical hyperalgesia and allodynia [9]. Furthermore, NGF injected into rat muscle tissue, increases the expression of NMDA receptors in the peripheral endings of muscle nociceptors, which sensitize the nerve ending [35]. Indeed, biopsy results from our group supports an up-regulation of NMDA receptors in the masseter muscle of healthy volunteers 3 days after NGF injection (Alhilou et al., in preparation). This could perhaps explain the mechanism on the NGF-induced allodynia observed also in this study.

Injection of 0.2 mL sterile solution of glutamate (1M) into the NGF pretreated masseter muscle caused spontaneous pain in the masseter muscle, lasting for 10 min with its peak shown around 90 s after the injection. The magnitude of the pain and its duration were similar to that reported in previous studies where glutamate was injected into the masseter muscle separately [25] which means that the glutamate injection did not induce any additional pain in the NGF sensitized muscle. This is also consistent with a previous study [12].

It has been shown that both systemic and local administration of glutamate causes mechanical sensitization in the muscle [6, 7], but similar to spontaneous pain there was no additional effect by glutamate on muscle sensitization induced by NGF in this study. This is also consistent with previous results from the masseter muscle [12]. The lack of additive effect by glutamate could be due to saturation of pain sensitization in the masseter muscle. Or, it could be a more centralized phenomenon where NGF induces descending pain modulation and thereby prevents further nociceptive signaling caused by glutamate. The latter would be consistent with a previous study in which hypertonic saline injection into the tibialis anterior muscle sensitized by NGF increased PPTs in men [13]. Even if insignificant, PPTs appear to be slightly increased after glutamate in the men also in this study. Nevertheless, higher concentration of glutamate was observed in the masseter muscle and in saliva of myofascial pain patients than pain-free participants which indicates that glutamate is one of the key substances to understand the pathophysiology of myalgia [36–38].

Higher pain and fatigue scores in women than men after a 1-min gum chewing task were shown 5 min after glutamate injection into the NGF pretreated masseter muscle in this study. This indicates that there was a significant sex difference in perceived sensation of pain and fatigue. This finding could perhaps be explained by a difference between women and men in the expression and activation of glutamate or NGF receptors related to pain. In many clinical and human experimental studies, sex differences in pain-related parameters have been reported. In the aforementioned animal study by Wong et al., the duration of NGF-evoked mechanical sensitization was longer in female rats [23]. In addition, the response to 2-amino-5-phosphonopentanoic acid, an NMDA receptor antagonist, reversed the mechanical sensitization only in male rats. Interestingly, another study showed that rats alter their chewing patterns in a sex-dependent manner when mechanical hyperalgesia is present in the masseter muscle [39]. Such sex differences in NGF induced mechanical sensitization could perhaps explain the sex difference in both perceived jaw functional limitations and pain and fatigue in response to chewing that was observed in this study. However, the mechanism behind this observation remains to be investigated.

This study has some strengths and limitations that should be considered. This is the first study to investigate sex difference in pain response to glutamate injection into NGF pretreated muscle. It could be considered a limitation of this study that emotional function was only assessed at baseline, since there is a possibility that the sex difference in self-reported pain perception observed in this study could be affected by the psychological status of the participants. Indeed, women scored higher in measures of anxiety (GAD-7), depression (PHQ-9), and unspecific physical symptoms (PHQ-15) at baseline, which might support this [40]. Therefore, repeated monitoring of psychological status could have captured such changes throughout the experimental period. On the other hand, this was not the primary aim of the study.

Taken together, the findings of this study further confirmed that glutamate does not augment NGF induced mechanical sensitization. However, pain and fatigue evoked by jaw function were higher in women after glutamate injection. This suggest that sex differences reported for TMD myalgia, mimicked by glutamate mediated pain in this study, may be greater for measures of perceived jaw function. This could be considered in clinical evaluation since pain that is worsened by jaw function is one of the key symptoms in TMD myalgia.

## Data Availability Statement

The raw data supporting the conclusions of this article will be made available by the authors, without undue reservation.

## Ethics Statement

The studies involving human participants were reviewed and approved by the local ethics committee (approval No. 1-10-72-199-15, Aarhus County, Denmark. The patients/participants provided their written informed consent to participate in this study.

## Author Contributions

AS participated in acquisition, analysis, and interpretation of the data, as well as drafting the manuscript. AA participated in data collection and revised the manuscript. PS contributed to interpretation of the data and participated in revision of the manuscript. ME participated in conception and design of this study and drafted the manuscript. NC conceived the study, participated in its design, and revised the manuscript. All authors read and approved the final manuscript.

## Conflict of Interest

The authors declare that the research was conducted in the absence of any commercial or financial relationships that could be construed as a potential conflict of interest.
